# Solution-Processed Polymer Dielectric Interlayer for Low-Voltage, Unipolar n-Type Organic Field-Effect Transistors

**DOI:** 10.1021/acsami.3c11285

**Published:** 2023-11-22

**Authors:** Andrea Perinot, Francesca Scuratti, Alberto D. Scaccabarozzi, Karolina Tran, Jorge Mario Salazar-Rios, Maria Antonietta Loi, Giovanni Salvatore, Simone Fabiano, Mario Caironi

**Affiliations:** † Center for Nano Science and Technology, Istituto Italiano di Tecnologia, Via Raffaele Rubattino 81, 20134 Milan, Italy; ‡ Photophysics and OptoElectronics, Zernike Institute for Advanced Materials, University of Groningen, Nijenborgh 4, 9747 AG Groningen, The Netherlands; § Department of Molecular Sciences and Nanosystems, Ca’ Foscari University of Venice, Via Torino, 155—Alfa Building, 30172 Mestre Venice, Italy; ∥ Laboratory of Organic Electronics, Department of Science and Technology, Linköping University, 60 174 Norrköping, Sweden

**Keywords:** organic electronics, organic transistors, field-effect transistors, low voltage, cross-linking, multilayer dielectric, doping

## Abstract

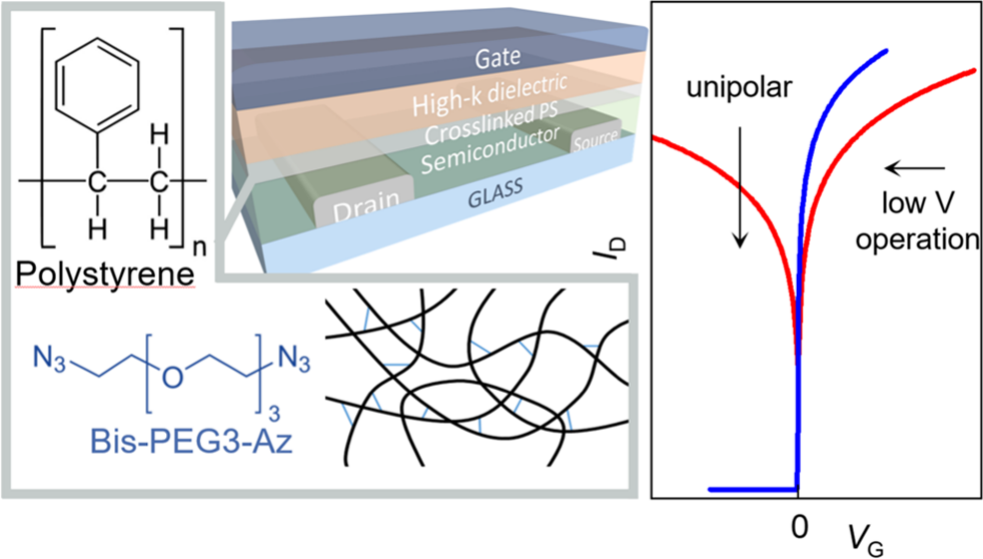

The integration of organic electronic circuits into real-life applications compels the fulfillment of a range of requirements, among which the ideal operation at a low voltage with reduced power consumption is paramount. Moreover, these performance factors should be achieved via solution-based fabrication schemes in order to comply with the promise of cost- and energy-efficient manufacturing offered by an organic, printed electronic technology. Here, we propose a solution-based route for the fabrication of low-voltage organic transistors, encompassing ideal device operation at voltages below 5 V and exhibiting n-type unipolarization. This process is widely applicable to a variety of semiconducting and dielectric materials. We achieved this through the use of a photo-cross-linked, low-*k* dielectric interlayer, which is used to fabricate multilayer dielectric stacks with areal capacitances of up to 40 nF/cm^2^ and leakage currents below 1 nA/cm^2^. Because of the chosen azide-based cross-linker, the dielectric promotes n-type unipolarization of the transistors and demonstrated to be compatible with different classes of semiconductors, from conjugated polymers to carbon nanotubes and low-temperature metal oxides. Our results demonstrate a general applicability of our unipolarizing dielectric, facilitating the implementation of complementary circuitry of emerging technologies with reduced power consumption.

## 1 Introduction

Organic electronics is an enabling technology with broad applications in industries like healthcare, design, energy, Internet of Things (IoT), and entertainment.^1^ The power of such technology results from the possibility of combining electronic functionalities with a set of remarkable advantages, such as flexibility, conformability, and biocompatibility and with the promise of low-cost and high-throughput manufacturability. In this respect, solution-based thin-film coating methods are attractive, combining the possibility of accessing established high-throughput fabrication techniques (e.g., printing, wide-area coating) with low-temperature processing.^2,3^ The potential of organic electronics has been proven more clearly in recent years through a range of proof-of-concept applications in the fields of healthcare, biomedical, and wearable and flexible smart surfaces.^4−8^

Nonetheless, further improvement of a set of performance factors is required to unlock the full potential of the technology and to enlarge the range of target applications, for example, for distributed stand-alone IoT devices. In particular, it is necessary to achieve power-efficient operation of the electronics with a low operational voltage (in the range of few volts) and low power consumption, as required for compatibility with flexible batteries^9^ or energy harvesters.^10−12^ Moreover, it is necessary to do so while adopting fabrication processes and materials that comply with the vision of low-cost manufacturing of end products.

To enable the implementation of CMOS-like architectures, strongly unipolar field-effect transistors (FETs) of both types (p- and n-) are necessary, as they can result in improved performance compared to unipolar circuit design, primarily manifested through lower power consumption, higher gains, and improved noise margins.^13^ However, a majority of organic semiconductor materials, including the most performing to date, are capable of conducting both holes and electrons in their pristine state and require specific architectures to yield an unipolar behavior. In the past, different approaches have been explored to this purpose: inhibiting the injection of one kind of charge carrier (e.g., through adoption of electrodes with appropriate workfunction or through electrode modification),^14^ introducing chemical species acting as traps,^15^ and shifting the Fermi energy with the introduction of molecular dopants^16,17^ or ordered dipoles at the dielectric interface.^18^ On the other hand, these methods are either not always easy to integrate with solution-processed architectures or present drawbacks (e.g., disruption of molecular order upon addition of dopants, interdiffusion of dopants, need for synthesis of appropriately modified materials). Moreover, the specific case of n-type unipolarization via the use of dopants is especially problematic due to the requirement of materials with very low ionization potential exhibiting high chemical reactivity.^19^

A second requirement for such devices is ideal low-voltage operation, which is driven by several factors: the semiconductor layer should feature a narrow density of states below the transport energy and a limited number of deep trap states within the bandgap,^20^ while the dielectric layer should yield high capacitance per unit area. The dielectric should also satisfy a number of further key requirements: high dielectric strength and low current leakage across the dielectric layer,^21^ an energetically smooth semiconductor–dielectric interface,^22^ a low number of trapping sites at the interface and in the bulk of the dielectric to prevent hysteresis and/or memory effects,^23,24^ and the absence of ferroelectricity and of dielectric relaxation at low frequency.^25^ Moreover, another fundamental ingredient to the ideal low-voltage operation of organic FETs (OFETs) is minimization of the contact resistance,^26,27^ which is usually achieved via self-assembled/charge-injection layers.^28^ In this regard, the adoption of top-gate, staggered architectures^29^ offers the highest flexibility toward the achievement of this goal.

Encompassing all of the aforementioned features in staggered architectures has been problematic, especially for top-gated architectures: high-*k* dielectrics can negatively affect charge transport,^22,30^ while solution-processed thin low-*k* dielectric layers are prone to suffer unacceptably high leakage current, unless specific fabrication routes are employed.^31^ Alternative solutions could in principle be constituted by ionic dielectrics (which are however limited by intrinsically low relaxation frequency,^21,32^ high OFF current, and/or hysteresis due to the semiconductor doping dynamics^33^) or polymer/nanoparticle composites^34−36^ (which generally exhibit reduced breakdown strength owing to local field-enhancement effects^25^). A more general approach consists in the adoption of a solution-processed multilayer stack, comprising a thin low-*k* dielectric layer in contact with the semiconductor to provide an optimal energetic landscape for efficient charge transport, capped with a high-*k* polymer dielectric, thus reducing the current leakage without strongly lowering the total areal capacitance.^33,37−42^ Such an approach is not free from complications since the fabrication of the stack requires a set of solvents both suitable for uniform, pinhole-free deposition of layers and orthogonal to the underlying material, thus adding further severe constraints. Previous approaches for the fabrication of solution-processed multilayered stacks included not only procedures based on solvent orthogonality^43^ but also methods relying on fluorinated dielectric polymers, which convey excellent insolubility against nonfluorinated solvents,^44^ or on the exploitation of the vertical segregation of low-*k*/high-*k* polymer blends into bilayers.^45^ However, these solutions are applicable only to specific choices of dielectrics and semiconductors. Moreover, solution-processed high-capacitance dielectric stacks for top-gate, staggered architectures operating at frequencies above 1 MHz (promoting the integration of wireless communication capabilities into this kind of devices) were rarely shown, also due to the lack of high-*k*, solution-processable dielectrics with sufficiently high relaxation frequency.^25^

Therefore, it is desirable to identify an approach for the implementation of solution- and low-temperature-processable gate dielectrics for top-gate, staggered low-voltage unipolar FETs general enough to be compatible with a variety of semiconductors, guaranteeing ideal device operation and promoting the unipolar behavior of the device.

Here, we illustrate a solution-based route for the fabrication of a dielectric layer yielding n-type unipolar FETs based on a variety of classes of semiconductor materials and correctly operating at a low voltage. The proposed approach relies on a multilayered dielectric architecture consisting of a thin cross-linked polystyrene layer and of different high-*k* dielectrics and is applicable to different semiconducting materials (polymer semiconductors, carbon nanotubes, metal oxides). FETs integrating such dielectrics operate ideally and without hysteresis at voltages below 5 V owing to areal capacitances as high as 40 nF/cm^2^ and reduced gate leakage current. Moreover, a dielectric relaxation frequency in excess of 1 MHz is achievable, enabling the use of this dielectric in high-frequency-oriented applications. In addition, such a dielectric possesses n-type unipolarizing capabilities deriving from the use of an azide-based cross-linker, enabling the fabrication of optimized complementary circuitry. Such structures can for example be easily integrated in reported multilayer architectures.^6,46,47^ Our results, which rely only on solution-based techniques and on commercially available materials, offer a viable route for the future low-cost mass production of low-voltage, efficient circuits for consumer applications.

## 2 Results and Discussion

### 2.1 Process and Materials

**Figure 1:**
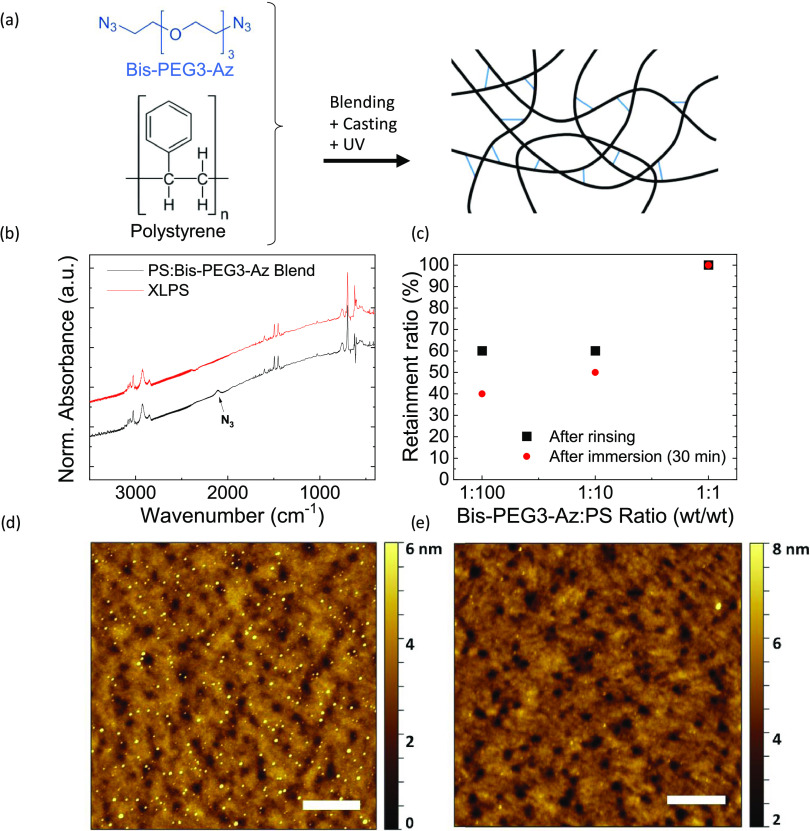
(a) Chemical structures of polystyrene and bis-PEG3-Az and schematic of the cross-linking process. (b) Normalized IR absorption spectra of the PS:bis-PEG3-azide blend and of the XLPS, (c) retainment ratio of cross-linked thin films at different bis-PEG3-Az/PS blending ratios after rinsing or 30 min immersion in toluene, and atomic force microscopy (AFM) topography of a 40 nm thick cross-linked film (bis-PEG3-Az/PS 1:10) deposited on top of P(NDI2OD-T2) before (d) and after (e) rinsing with *n*-butyl acetate (scale bars: 1 μm).

Our approach for low-voltage, unipolar, solution-processed OFETs relies on the selection and design of a specific low-*k* dielectric interlayer to be integrated into multilayered dielectric stacks. We select polystyrene (PS, Figure 1a) as the main component of the interlayer since its suitable properties and general applicability as a low-cost dielectric material for FETs have been extensively demonstrated.^42,44,48−51^ We then combine the advantages offered by PS with the improved robustness provided by cross-linking, which includes a reduced tendency of the film to dewet upon thermal treatments,^52,53^ increased glass transition temperature,^54,55^ improved resistance of the film upon further solvent processing on top, and reduced current leakage.^56^ In particular, since thin polymer layers deposited on top of commonly used polymer semiconductors are prone to dewetting upon thermal annealing, photo-cross-linking approaches constitute an advantageous choice since the associated chemical reaction can be activated at moderate or room temperature. We choose a cross-linking approach based on nitrene chemistry, which relies on the photoinduced activation of an azide moiety to generate a highly reactive singlet nitrene that undergoes a C–H insertion reaction into the backbone of the PS molecules, which in turn cross-links to create an insulating network (Figure 1a). In previous works, such an approach was adopted to create similar cross-linked layers of dielectrics or semiconducting polymers by synthesizing azide-modified materials^57,58^ or azide-bearing small-molecule additives.^59−62^ Here, to simplify the adoption of such a process, we do not resort to the synthesis of custom azide-bearing molecules and we select the commercially available 1,11-diazido-3,6,9-trioxaundecane (bis-PEG3-Az, Figure 1a) as a small molecular additive. Our process is arranged as follows: we first blend appropriately concentrated solutions of PS and Bis-PEG3-Az in different ratios, and then we deposit a thin layer of this dielectric (25–150 nm, depending on the particular experiment) on the desired substrate (or the functional material, when fabricating the devices). The resulting dried layer is then exposed to UV radiation (λ = 254 nm) in a nitrogen atmosphere for 30 min.

### 2.2 Properties of the Cross-Linked Interlayer

We first tested the occurrence of the cross-linking reaction by measuring the IR spectra of different samples (Figure 1b): the absorption peak located near 2100 cm^–1^, which is attributed to azide stretching, disappears upon irradiation with UV for 30 min. Moreover, we tested the resistance of the cross-linked polystyrene (XLPS) interlayer to further solvent-based processing by measuring its retainment ratio (i.e., the ratio between the initial thickness of the layer and the thickness after solvent processing) after simple washing with a good solvent (toluene) and after a 30 min long immersion of the sample in the same solvent. The results (Figure 1c) highlight that the retainment ratio upon rinsing is always over 60% for all of the investigated polymer/linker blends, reaching up to 100% in the best case, and that cross-linking provides good solvent resistance even upon a more aggressive 30 min long immersion process. This result indicates that our interlayer is effectively rendered insoluble by the cross-linking and is able to withstand further solvent processing on top as required for the fabrication of functional bilayered dielectric stacks. In addition, to serve as a suitable dielectric interlayer for our device architecture, the PS layer requires the absence of pinholes when deposited on top of the semiconductor, to effectively decouple the underlying device channel from the top, high-*k* dielectric. We exemplarily tested our thin (40 nm) interlayer on top of the widely studied and good electron transporting polymer semiconductor poly[*N*,*N*′-bis(2-octyldodecyl)-naphthalene-1,4,5,8-bis(dicarboximide)-2,6-diyl]-alt-5,5′-(2,2′-bithiophene) P(NDI2OD-T2), analyzing the morphology of the top surface through AFM imaging before and after washing it with *n*-butyl acetate, a good solvent for polystyrene (Figure 1d,e). The dielectric surface presents the same surface roughness of 0.7 nm rms before and after washing, and despite the film topography showing few valley-like irregularities with an average depth of ∼3 nm, the surfaces of our dielectric do not present through holes in both cases (see the Supporting discussion in the Supporting Information).

We investigated the electrical properties of XLPS also in terms of the dependence of the dielectric constant ε_r_ vs frequency and applied electric field (see the Supporting discussion in the Supporting information). We determined that the cross-linking process does not introduce any evident nonideality, the frequency response of ε_r_ is flat across all of the investigated range of frequencies (10^2^–10^7^ Hz) with no appreciable dependence on the applied electric field (0–500 kV/cm), and the measured value of ε_r_ = 2.6 is consistent with the dielectric constant of pristine polystyrene.

### 2.3 Dielectric Properties of High-*k* Polymers

In addition to a robust low-*k* interlayer, multilayer high-capacitance dielectric stacks require a high-*k* material on top to prevent the appearance of high leakage current in FET devices while ensuring a low-voltage operation. The required properties of such material include (1) high permittivity with a sufficiently flat behavior within an appropriately wide frequency range and (2) no variation of the dielectric constant of the material with the applied electric field. We selected a variety of high-*k* solution-processable polymers (i.e., cyanoethylated pullulan (CEP), poly(vinylidenefluoride–co-trifluoroethylene) (PVDF–TrFE), poly(vinyl alcohol) (PVA), poly(vinylidenefluoride–trifluoroethylene–chlorofluoroethylene) (PVDF–TrFE–CFE), and poly(vinylidenefluoride–trifluoroethylene–chlorotrifluoroethylene) (PVDF–TrFE–CTFE)), and we measured the frequency behavior of their dielectric constant at different applied electric field (see the Supporting discussion in the Supporting information). We have determined that the investigated materials exhibit ε_r_ in the range from 8 to 13 and most of them feature a nearly flat dependence of ε_r_ vs frequency up to their respective dielectric relaxation frequency and negligible ferroelectric effects. In particular, CEP shows ε_r_ ∼ 13 (at 100 Hz), and its dielectric relaxation frequency (here, defined as the frequency corresponding to an attenuation of 3 dB of ε_r_ with respect to its value at 1 kHz) is 1.73 MHz.

### 2.4 Electrical Properties of Dielectric Stacks

We have then fabricated metal–insulator–metal (MIM) structures comprising the full multilayer dielectric stacks using the different high-*k* polymers and evaluated the behavior of the capacitance vs frequency and vs applied voltage, as well as the current leakage per unit area (Figure S1 and Table 1). The areal capacitances of the realized devices span between 23 and 38 nF/cm^2^ (at 100 Hz), with no appreciable variation with the average applied electric field in the range of interest (0–10 V). The lowest current leakage is achieved for the stack based on PVA, with less than 1 nA/cm^2^ at an applied voltage of 10 V. The current leakage lies below 1 μA/cm^2^ at 10 V for all of the investigated stacks and in the order of 1 nA/cm^2^ at 5 V.

**Table 1:** Summary of the Electrical Figures of Merit for the Dielectric Stacks Based on XLPS and Different High-*k* Polymers

stack composition	ε_r_ (high-*k* polymer, at 100 Hz)	areal capacitance (at 100 Hz) [nF/cm^2^]	areal current leakage (at 5 V) [nA/cm^2^]
XLPS (40 nm)/CEP (110 nm)	13	37.5	4.3
XLPS (40 nm)/PVDF–TrFE (160 nm)	8.7	34.9	1.4
XLPS (40 nm)/PVA (140 nm)	8.1	23.3	0.5
XLPS (40 nm)/ PVDF–TrFE–CFE (200 nm)	8.9	23.5	0.5
XLPS (40 nm)/ PVDF–TrFE–CTFE (300 nm)	17.2	25	14.3

**Figure 2:**
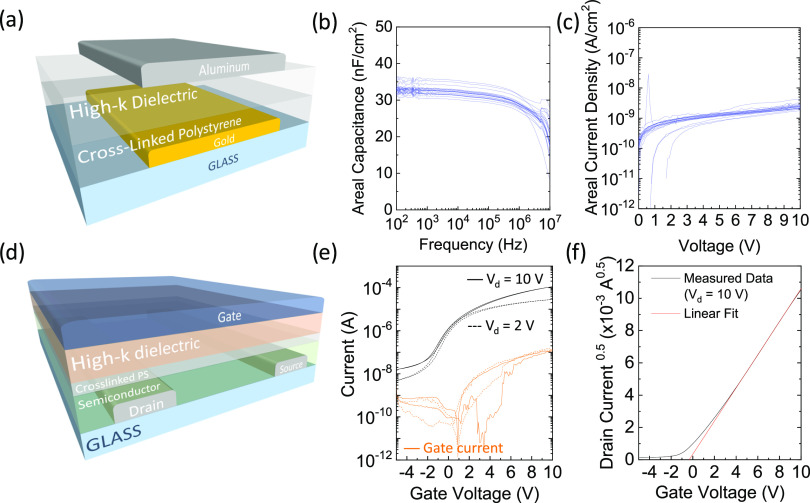
(a) 3D view of the fabricated MIM structures, (b) areal capacitance and (c) leakage current density for a set of 20 MIM structures integrating a bilayer dielectric with 40 nm thick XLPS and 110 nm thick CEP layers, (d) 3D view of the OFET structure, (e) transfer curve for the OFET based on our bilayer dielectric with a CEP layer thickness of 150 nm (areal capacitance *C*_diel_ = 32.9 nF/cm^2^), and (f) the transfer curve for the same device (*V*_d_ = 10 V) in a linear scale.

The stack based on CEP exhibits the best performance overall: its high dielectric constant ε_r_ ∼ 13 allows one to reach a high capacitance in excess of 37 nF/cm^2^ in stacks with a total thickness of ∼150 nm. Despite such a low thickness, the areal leakage current is below 10 nA/cm^2^ for an extended voltage range (up to 8–9 V), suitable for the fabrication of high-performance transistors. In addition, the roll-off of ε_r_ with the increase of the bias frequency is negligible until ∼1 MHz, which allows for high-frequency operation of low-voltage, solution-processed organic FETs. Thus, we selected a bilayer dielectric configuration including a 40 nm thick XLPS layer and a 110 nm thick CEP layer (Figure 2a) and tested the repeatability of the areal capacitance and leakage current density over a set of 20 MIM structures. The measured areal capacitances lie between 31 and 37 nF/cm^2^ (Figure 2b), while the leakage current density is always below 4 nA/cm^2^ (Figure 2c) at a voltage of 10 V (corresponding to an average electric field of 0.67 MV/cm). The average areal capacitance of these capacitors is 33 nF/cm^2^ at 100 Hz with a standard deviation of 1.4 nF/cm^2^, while the average areal leakage current is 1.2 nA/cm^2^ at 5 V with a standard deviation of 0.2 nA/cm^2^. These results reveal an optimal lab-scale replicability of the performance of such structures and suggest their suitability for low-voltage FET operation.

### 2.5 Electrical Properties of FETs and the Semiconductor/Dielectric Interface

To test the electrical quality of the interface between the cross-linked interlayer and polymer semiconductors, we fabricated top-gate, staggered OFETs using P(NDI2OD-T2) (Figure 2d). We adopted a dielectric stack composed of 40 nm thick XLPS and a 150 nm thick CEP layer, and we show in Figure 2e the measured transfer curve for a device with channel length *L* = 10 μm and channel width *W =* 2 mm. Such a curve demonstrates ideal operation of the device at gate voltages smaller than 10 V, both in the linear (*V*_d_ = 2 V) and in the saturation (*V*_d_ = 10 V) regimes, while the gate current leakage is limited to a value 3 orders of magnitude below the maximum ON current. The subthreshold slope for such a device reaches a maximum value of 1 V/dec. The square root of the saturation drain current vs gate voltage is correctly linear (Figure 2f), and by linearly fitting such a curve, we extract a threshold voltage *V*_t_ = −0.34 V and an apparent saturation charge mobility μ_app_ = 0.32 cm^2^/V/s. We also plot in Figure S2a the calculated electron mobility vs gate voltage in both operation regimes: in the linear regime, the curve is completely flat, while in the saturation regime, a mild gate-bias dependency is highlighted. Correct transistor operation and low gate leakage can also be achieved with a maximum bias voltage of 5 V (Figure S2b). To verify that the thin XLPS interlayer is effective in fully decoupling any influence of the top high-*k* polymer on charge transport, we fabricated another FET with the same geometry and structure and with a thicker XLPS layer (60 nm). If any detrimental effect of the top dielectric layer were affecting charge transport, the increase of the separation between the channel and such layer would improve the transfer characteristics of the OFET. The device correctly operates with a performance similar to the device with a thinner XLPS layer (Figure S3a). We compare the two devices by plotting the measured drain current (normalized to the areal capacitance of the two different dielectric stacks) vs *V*_g_*– V*_t_ (Figure S5b, black and red lines). The two curves are perfectly superimposed (excluding the OFF-current region), highlighting that no improved charge transport is achievable by increasing the separation between the channel and top dielectric and revealing that an interlayer thickness of 40 nm is sufficient to shield the effect of traps or dipoles of the high-*k* polymer. On the other hand, when thinning down the interlayer thickness to 25 nm, anticlockwise hysteresis appears in the transfer curves (Figure S4). In general, such an effect can be attributed to ferroelectric polarization and/or charge injection from the gate into the dielectric.^23^ Indeed, both explanations are consistent with the appearance of hysteresis as the electric field across the CEP layer (*E*_CEP_) increases alongside the reduction of the thickness of the XLPS layer. By calculating the magnitude of *E*_CEP_ for the different thicknesses of the XLPS layer for a gate bias of 10 V (see the Supporting discussion in the Supporting information), a maximum value for *E*_CEP_ = 364 kV/cm is obtained, well below any threshold for the (possible) appearance of ferroelectric effects (see Figure SD1). This remark rules out the latter reason as the origin of hysteresis while suggesting that non-negligible charge injection from the gate might take place in such structures for a sufficiently high electric field. In addition, the calculation of the magnitude of the electric field across the XLPS layer (*E*_XLPS_) highlights that in the case of the thinnest XLPS layer of 25 nm, *E*_XLPS_ ≈ 2 MV/cm (Table SD1), close to the breakdown strength of thin films of polystyrene and, more generally, of other commonly adopted polymer dielectrics.^63^ The use of thin polymer layers of this kind, when the overall structure induces such a high electric field across the layer, might not be sufficient to prevent the injection of charge carriers from the semiconductor to the interface (or bulk) of the high-*k* polymer and might lead to nonidealities (e.g., memory effects, hysteresis). In agreement with these observations, we concluded that an XLPS layer with a thickness not lower than 40 nm is necessary for the correct operation of FETs.

After fixing the optimal XLPS layer thickness to 40 nm, we fabricated OFETs integrating top CEP layers of 110 and 80 nm to assess the possibility of increasing the overall stack capacitance. Both devices correctly operate at a maximum voltage of 10 V, while the gate current leakage is kept 2–3 orders of magnitude below the ON current (see Figure S5a for the device with a CEP thickness of 80 nm). Also in this case, when comparing the measured drain current (normalized to the areal capacitance of the two different dielectric stacks) vs *V*_g_*– V*_t_ (Figure S5b), we observe the correct superposition of the curves for the different devices, highlighting that the FET performance is preserved with the scaling of the dielectric thickness.

### 2.6 N-Type Doping Promoted by the XLPS Interlayer

**Figure 3:**
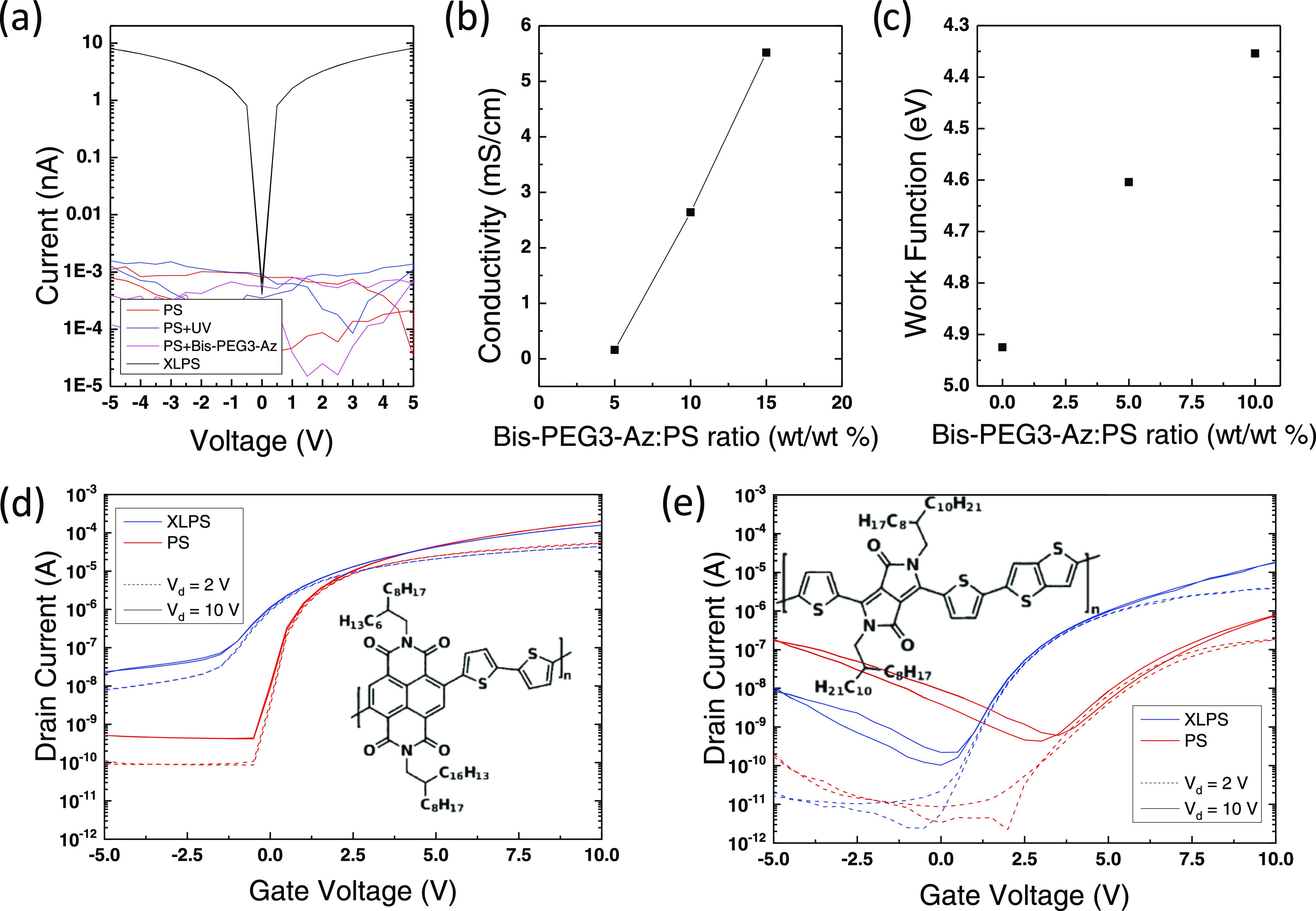
(a) Measured conductivity of P(NDI2OD-T2) films capped with different versions of our dielectric interlayer, (b) conductivity of n-doped P(NDI2OD-T2) films vs dopant concentration within the XLPS interlayer, (c) workfunction of n-doped P(NDI2OD-T2) films vs dopant concentration within the XLPS interlayer, and transfer curves of (d) n-type FETs (*W* = 2 mm, *L* = 10 μm) and (e) p-type FETs (*W* = 2 mm, *L* = 5 μm) with the XLPS/CEP or pristine PS/CEP dielectric.

The FETs exhibit an increased OFF current compared to similar architectures with the same semiconductor and more conventional dielectric layers, based, for example, on poly(methyl methacrylate).^64^ Thus, we further investigated the role of XLPS in such an increase. We measured the conductance *G* of a set of samples consisting of a layer of P(NDI2OD-T2), capped by different versions of our dielectric interlayer (Figure 3a). First, we confirmed that the conductance of the semiconducting layer capped with pristine polystyrene is negligible. We also did not detect any appreciable change of *G* upon exposure of this sample to UV for 30 min, clarifying that no degradation of P(NDI2OD-T2) and/or polystyrene occurs due to the photoexposure. We then mixed bis-PEG3-Az and polystyrene in a ratio of 1:10 and used such blend to cap the layer of the semiconductor. No increase in the conductance of P(NDI2OD-T2) is detected due to the presence of nonexposed bis-PEG3-Az. Instead, when the dielectric polymer is exposed to UV and cross-linked, we measure a significant increase in *G*, suggesting that the combination of the azide-based cross-linker and UV treatment induced n-doping of the semiconductor. We also verified that such a doping effect is not activated by heat, up to a temperature of 120 °C. We then varied the concentration of bis-PEG3-Az in the blend between 5 and 15%, and we identified a linear trend of the measured conductivity with the increase of the cross-linker concentration (Figure 3b). We then measured the workfunction of P(NDI2OD-T2) films after coming into contact with our XLPS interlayer, by delaminating the latter prior to measurement. The pristine semiconductor exhibits a workfunction close to 4.9 eV (Figure 3c). The workfunction for the P(NDI2OD-T2) films, after being interfaced with XLPS, decreases with an increasing concentration of bis-PEG3-Az in the interlayer, in agreement with an increased electron density.

In order to confirm our observations suggesting a doping effect on the semiconductor upon contact with XLPS, we further investigated such an effect in FET structures with two different semiconductors. We first compared the previously fabricated n-type transistors based on P(NDI2OD-T2) and the dielectric stack with XLPS and CEP with a second version of the full dielectric stack, realized with pristine PS and CEP. The two stacks are realized with the same thicknesses and thus areal capacitances. A doping effect is revealed by such comparison (Figure 3d): while the PS-based FET exhibits correct operation and OFF currents below the nA, the XLPS-based device exhibits increased OFF current and a decrease of the steepness of the curve in the subthreshold regime. We then fabricated FETs employing the semiconducting polymer DPP–TTT as the active layer while retaining the same dielectric stacks. DPP–TTT devices are indeed known to exhibit strongly ambipolar transport in TGBC configuration with Au source–drain electrodes.^65,66^ Also in this case, a clear n-type doping effect is detected in the device integrating XLPS compared to the one with the pristine interlayer, as highlighted by the decrease of the threshold voltage for n-type operation, by the increase of the ON current, and by a steeper subthreshold slope (Figure 3e). In practice, our dielectric stacks based on XLPS promote the n-type unipolarization of organic semiconductors via an n-doping effect, an effect that can be exploited, for example, for the realization of low-power complementary circuitry exhibiting improved figures of merit.^17,67^ In future implementations, the cross-linker concentration can be tuned according to the necessary doping level for the semiconductor in use or the application specifications.

Despite the observation of clear n-doping upon cross-linking with bis-PEG3-Az, the underlying doping mechanism is still elusive. Nevertheless, as we mentioned earlier, the cross-linking involves the activation of an azide moiety to generate a highly reactive singlet nitrene that leads the molecule to link two PS chains. We speculate that upon cross-linking, the photoproduct of bis-PEG3-Az forms an amine. The NH has an electron pair that is capable of n-doping through electron transfer, similarly to the doping mechanism of many amine-based n-dopants, such as PEI.^17^ Nevertheless, the actual mechanism might be different and undergo the formation of intermediate products and/or complex reactions.

### 2.7 General Applicability of the Approach for a Variety of Materials

**Figure 4:**
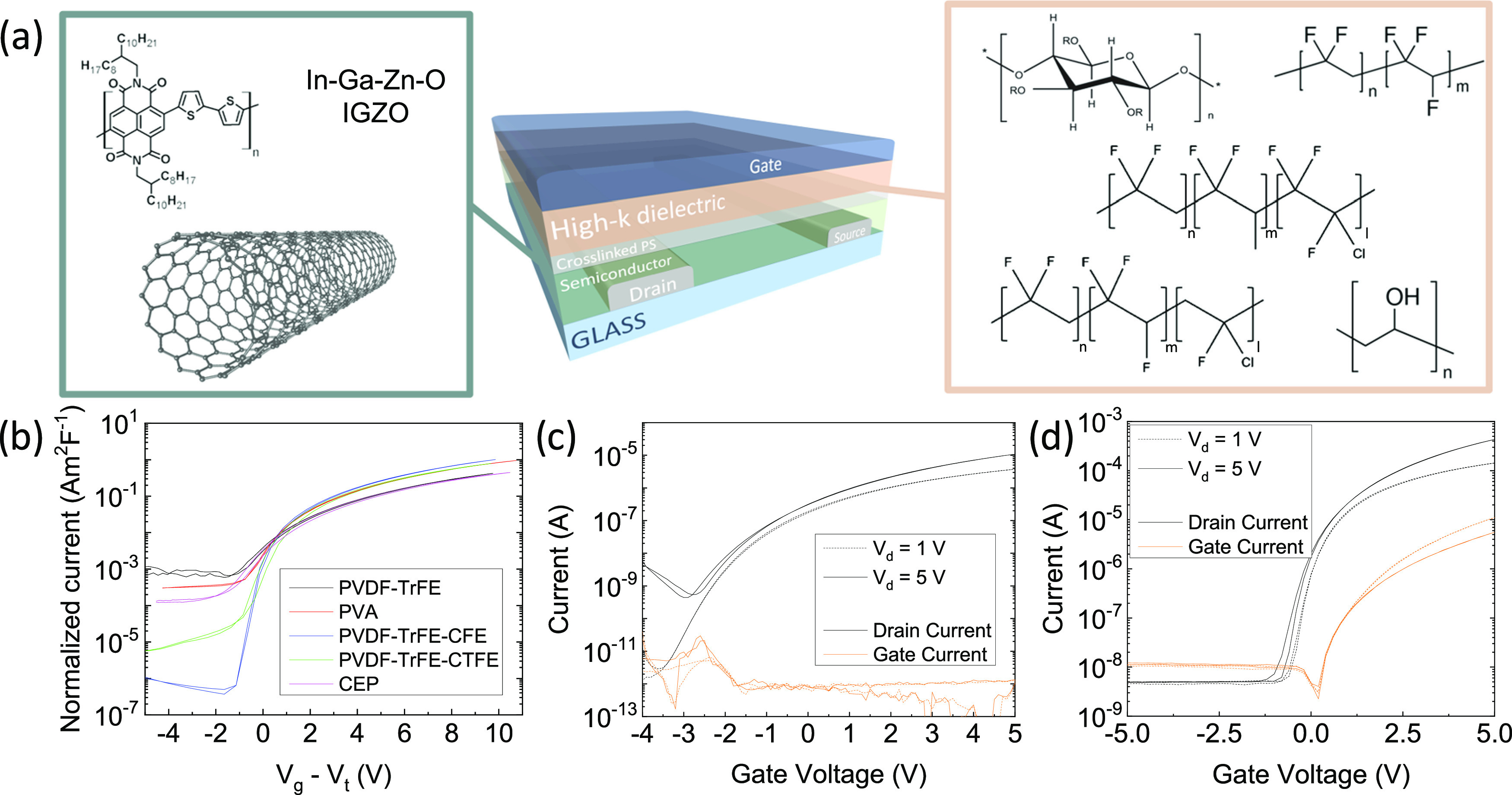
(a) 3D view of the realized FET structure, with indication of the different semiconductor materials and high-*k* polymers used; (b) transfer curves in the saturation regime for FETs (*W* = 2 mm, *L* = 10 μm) based on P(NDI2OD-T2), XLPS (40 nm), and different high-*k* polymers: CEP (80 nm), PVDF–TrFE (160 nm), PVA (140 nm), PVDF–TrFE–CFE (200 nm), and PVDF–TrFE–CTFE (300 nm); (c) transfer curve for an FET (*W* = 200 μm, *L* = 20 μm) based on polymer-wrapped carbon nanotubes, XLPS (40 nm), and CEP (110 nm); and (d) transfer curve for an FET (*W* = 2 mm, *L* = 20 μm) based on IGZO, XLPS (40 nm), and PVDF–TrFE (100 nm).

Our approach is general and can be implemented with a variety of choices for high-*k* dielectric materials and the semiconductors. We first tested a set of low-voltage OFETs based on P(NDI2OD-T2) and high-capacitance dielectric stacks integrating our XLPS interlayer and the different high-*k* polymers mentioned earlier in the text (Figure 4a).

The normalized drain current vs *V*_g_*– V*_t_ (Figure 4b) highlights the correct operation of the FETs for the whole set of high-*k* polymers. Some differences can be identified in terms of the OFF current, which can be attributed to a variable efficiency of the doping effect of bis-PEG3-Az in the different versions of the dielectric stack. However, even considering this effect, our data show clearly that our XLPS interlayer enables correctly operating low-voltage FETs integrating a variety of different high-capacitance, solution-processed dielectric stacks.

The generality of our approach is not limited to the choice of the high-temperature dielectric material. We further demonstrated that our approach is also applicable with a variety of different semiconductors in addition to polymers. We selected polymer-wrapped semiconducting carbon nanotubes (CNTs) and indium gallium zinc oxide (IGZO) as representative examples for the categories of carbon-based and metal-oxide semiconductors. The measured transfer curve of an FET integrating CNTs and a CEP-based dielectric stack shows proper low-voltage operation at a maximum bias of 5 V (Figure 4c), with extremely reduced gate leakage. The maximum extracted charge mobility for this device is ∼3 cm^2^/(V s) (Figure S6a), while the transfer curve also shows the correct linearity of the square root of the current vs *V*_g_ (Figure S6b). Similarly to the unipolarization effect previously highlighted for polymer semiconductors, also here, the doping effect induced by the XLPS interlayer switches the behavior of CNT-based transistors from ambipolar to n-type unipolar. The comparison with the transfer curve measured on an FET with the same CNTs and a conventional PMMA layer (*C*_diel_ = 5.8 nF/cm^2^, Figure S7) highlights that p-type transport is suppressed, while n-type operation is enhanced in terms of electron mobility (3 cm^2^/(V s) vs 1.7 cm^2^/(V s) in the linear regime for the doped and undoped case, respectively). The unipolarization promoted by our cross-linker-induced n-type doping yields performances comparable to other unipolarization techniques already used in the literature for solution-processed CNT-based FETs.^68,69^ Despite being challenging to unambiguously define the doping mechanism induced by the cross-linker in such a complex multicomponent system, considering the doping effect observed for P(NDI2OD-T2) and DPP–TTT, we speculate that Bis-PEG3-Az leads to the doping of the polymer-wrapped component rather than to the CNT directly. Indeed, the charge transport in the polymer-wrapped CNT is known to be highly sensitive to the interplay between the CNT network and the wrapping polymer, with tremendous effects on the transport.

Analogously, the low-voltage FET based on IGZO and a dielectric stack with PVDF–TrFE correctly operates with a sharp turn-on and an ON–OFF ratio of ∼10^5^, despite a higher gate leakage compared to the other semiconductors (Figure 4d). The extracted mobility for such a device is as high as 10 cm^2^/(V s) both in the linear and saturation regimes (Figure S8a), in agreement with the performance measured by other groups.^70,71^ Moreover, the superior features of such a semiconductor in terms of narrow DOS enable a sharp increase of the current in the subthreshold region and correct operation down to a gate bias of 1 V (Figure S8b).

## 3 Conclusions

In conclusion, we have shown that a solution-based route for the fabrication of polymeric high-*k*/low-*k* dielectric stacks with a high capacitance (up to 40 nF/cm^2^) and low leakage current density (below 1 nA/cm^2^) for low-voltage operation of n-type FETs is feasible. The key enabler of this approach is a thin low-*k* dielectric interlayer based on cross-linked polystyrene, which forms a suitable interface with different classes of semiconductors, both organics and inorganics compatible with large-area electronics, while at the same time providing good decoupling between the device channel and the high-*k* material. These features are fundamental to combine optimal charge transport properties in the FET channel and ideality of the device behavior with high flexibility in terms of the choice of the high-*k* dielectric and of the semiconductor materials. Moreover, our fabrication scheme is fully solution-based and is thus suitable for future implementation in mass-production facilities. Finally, our approach enhances the performance of n-type devices and improves their unipolarity due to the n-doping effect introduced by the adopted bis-PEG3-Az cross-linker. Such an effect promotes the realization of complementary circuits exhibiting reduced power consumption also with the use of ambipolar, low-bandgap semiconductors. The achievement of such a combination of features with a simple, solution-based method represents a valuable advancement toward the future implementation of performing circuitry for low-cost, mass-produced distributed electronic applications.

Indeed, the simplicity and effectiveness of our fabrication route allow easy integration in the fabrication of CMOS-like architectures. Indeed, bis-PEG3-Az can be potentially patterned, leading to unipolar n-type devices and unaltered p-type operation of suitably fabricated devices on the same substrate. Alternatively, multistack transistors can be fabricated, in which p-type devices are fabricated onto n-type devices. Finally, our approach can be potentially applied to p-type doping upon investigation of appropriate cross-linkers to achieve similar synergistic cross-linking/doping effects that we developed through the employment of bis-PEG3-Az.

### 3.1 Methods

#### 3.1.1 Materials

P(NDI2OD-T2) was purchased from Polyera, bis-PEG3-Az was purchased from Sigma-Aldrich and from Santa Cruz Biotechnology, polystyrene (*M*_w_ 2 000 000) and PVA were purchased from Sigma-Aldrich, CEP was purchased from Shin-Etsu Chemical, PVDF–TrFE was purchased from Solvay, PVDF–TrFE–CFE and PVDF–TrFE–CTFE were purchased from PolyK Technologies, and DPP–TTT was purchased from 1-Material (Product OS0300). Polymer-wrapped carbon nanotube ink consists of commercially available HiPCO single-walled carbon nanotubes (Unidym Inc.) functionalized with poly(3-dodecyltiophene-2,5-diyl) (P3DDT) in oDCB. A detailed solubilization procedure can be found in our previous works.^72^ 15 nm of IGZO was RF-magnetron sputtered at room temperature. The substrates consisted of low-alkali 1737F Corning glass.

#### 3.1.2 Solutions and Deposition Process

Polystyrene is dissolved in *n*-butyl acetate at concentrations between 5 and 10 g/L, bis-PEG3-Az is dissolved in *n*-butyl acetate at concentrations between 1.5 and 80 g/L, PVA is dissolved in water at a concentration of 25 g/L, CEP is dissolved in acetonitrile at concentrations between 15 and 25 g/L, PVDF-TrFE is dissolved in *n*-butyl acetate at a concentration of 30 g/L, PVDF–TrFE–CFE and PVDF–TrFE–CTFE are dissolved in *n*-butyl acetate at a concentration of 40 g/L, P(NDI2OD-T2) is dissolved in toluene at a concentration of 5 g/L, DPP–TTT is dissolved in dichlorobenzene at a concentration of 10 g/L, and CNTs are solubilized in oDCB. All of the polymer films excluding PVA and DPP–TTT are deposited via spin-coating in a nitrogen atmosphere. P(NDI2OD-T2) is deposited via off-centered spin-coating as in ref (64), DPP–TTT is deposited via bar-coating as in ref (73). The XLPS and pristine polystyrene interlayers are deposited via spin-coating at 1500 rpm for 5 min. All of the high-*k* interlayers are deposited via spin-coating at 1500 rpm for 2 min. CNTs were deposited by inkjet printing through a custom system, Jetlab 4xI-A, provided with a nozzle with an orifice diameter of 60 μm. Three printing passes were performed in order to obtain a well interconnected network.

#### 3.1.3 Cross-Linking

The deposited layer of a blend of PS and bis-PEG3-Az is exposed to UV radiation (254 nm) from a compact 4 W lamp (UVP UVGL-25), positioned at a distance of 2 cm from the sample, for 30 min in a nitrogen atmosphere.

#### 3.1.4 Fourier Transform Infrared (FTIR) Characterization

The samples were analyzed with a Bruker Vertex 70 FTIR spectrometer. Thin films (∼400 nm) of a blend of polystyrene and bis-PEG3-Az in a ratio of 10:1 were deposited on intrinsic silicon. The IR spectra of the UV-exposed thin film and that of the unirradiated thin film were measured. The measured spectrum of the silicon substrate was subtracted from that of the samples.

#### 3.1.5 MIM Capacitor Fabrication

A bottom electrode of gold (with a 1.5 nm thick adhesion layer of chromium) is deposited via thermal evaporation. In the case of single dielectric, the layer is deposited on top and then annealed for 20 min at 60 °C. For multilayer stacks, XLPS is deposited and cross-linked, then the high-*k* material is deposited, and the sample annealed for 20 min at 60 °C. Then, an aluminum top electrode is deposited via thermal evaporation. The area of the MIM capacitor is then measured using an optical microscope.

#### 3.1.6 FET Fabrication

Gold source and drain electrodes (with a 1.5 nm thick adhesion layer of chromium) were realized via standard photolithography and thermal evaporation. The realized patterns feature a channel width of 2 or 0.2 mm and a channel length of 20, 10, or 5 μm. The used geometries are specified in the text case by case. The semiconductors are deposited according to the procedures above and then processed as follows: P(NDI2OD-T2) is annealed in a nitrogen atmosphere for 1 h at 100 °C, DPP–TTT is annealed in a nitrogen atmosphere for 15 min at 100 °C, and the CNT films are annealed at 150 °C for 1 h. Then, XLPS or PS is deposited and cross-linked if necessary. After cross-linking, the high-*k* polymer is deposited, and the sample is annealed at 60 °C for 20 min. Then, aluminum gates are deposited via thermal evaporation. Before measurement, for P(NDI2OD-T2) and PVDF–TrFE dielectrics, the samples are annealed in a nitrogen atmosphere at 70 °C for 24 h, for P(NDI2OD-T2) and the other dielectric materials, the samples are annealed in a nitrogen atmosphere at 120 °C for 8 h, and for IGZO, the samples are annealed in a nitrogen atmosphere at 70 °C for 24 h.

#### 3.1.7 Conductivity Characterization

Gold electrodes with a length of 1 cm are deposited via thermal evaporation, separated by a distance of 6 mm. P(NDI2OD-T2) is deposited and annealed at 100 °C for 20 min. Pristine polystyrene or appropriate blends of PS and bis-PEG3-Az are deposited on top and exposed to UV if required. The samples are kept in a nitrogen atmosphere and never exposed to air (excluding a short time to allow for Kelvin probe measurement). The trends of conductivity vs dopant concentration were characterized using patterns consisting of interdigitated gold electrodes with a total length of 2 mm, separated by a gap of 20 or 10 μm. Contact resistance was accounted for by using the transmission-line method.

#### 3.1.8 Kelvin Probe Characterization

P(NDI2OD-T2) is deposited on a gold-coated glass substrate and annealed at 100 °C for 20 min. Appropriate blends of PS and bis-PEG3-Az are deposited on top and exposed to UV if required. The capping dielectric is delaminated using tape. The samples are kept in a nitrogen atmosphere and never exposed to air (excluding a short time to allow for sample loading). The measurement is performed using a KPTechnology KP020 system in a nitrogen-filled cabinet.

#### 3.1.9 Additional Characterization Instrumentation

The topography of the films was measured with an Agilent 5500 atomic force microscope operated in acoustic mode. The capacitance–frequency measurements are obtained via a Agilent 4294A impedance analyzer. The transfer curves of the FETs and the current–voltage characteristics of the samples were measured via an Agilent B1500A semiconductor parameter analyzer, while the samples are kept in a nitrogen atmosphere.

## Supporting Information Available

Capacitance–frequency measurements for MIM structures integrating multilayer dielectric stacks; transfer curve and calculated mobility in the linear and saturation regimes for an OFET based on P(NDI2OD-T2), CNTs, and IGZO; IR raw absorption spectra of the PS:bis-PEG3-azide blend and of XLPS; and supporting discussion on the frequency behavior of the high-*k* polymers and of cross-linked PS, on the magnitude of the electric fields in the dielectrics; and on the AFM analysis of cross-linked polystyrene before and after solvent washing (PDF)
