# Supplementary material for: Solution-Processed Polymer Dielectric Interlayer for Low-Voltage, Unipolar n-Type Organic Field-Effect Transistors
Source: ACS Appl Mater Interfaces. 2023 Nov 22;15(48):56095–105. doi: 10.1021/acsami.3c11285 (PMC10711709; doi:10.1021/acsami.3c11285)
Supplement: Supplementary file 1 — am3c11285_si_001.pdf [file am3c11285_si_001.pdf]

SUPPORTING INFORMATION

A Solution-processed Polymer Dielectric Interlayer For Low-voltage, Unipolar n-type Organic Field-effect Transistors

Andrea Perinot[1], Francesca Scuratti[1], Alberto D. Scaccabarozzi[1], Karolina Tran,[2] Jorge Mario Salazar-Rios,[2] Maria Antonietta Loi,[2] Giovanni Salvatore,[3] Simone Fabiano,[4] Mario Caironi[1,]*

[1] Center for Nano Science and Technology, Istituto Italiano di Tecnologia, via Raffaele Rubattino 81, Milan, Italy

[2] Photophysics and OptoElectronics, Zernike Institute for Advanced Materials, University of Groningen, Nijenborgh 4, Groningen, The Netherlands

[3] Department of Molecular Sciences and Nanosystems, Ca' Foscari University of Venice, Via Torino, 155 - Alfa Building, 30172 Mestre Venice, Italy

[4] Laboratory of Organic Electronics, Department of Science and Technology, Linköping University, Norrköping, Sweden.

[*] mario.caironi@iit.it

SUPPLEMENTARY FIGURES

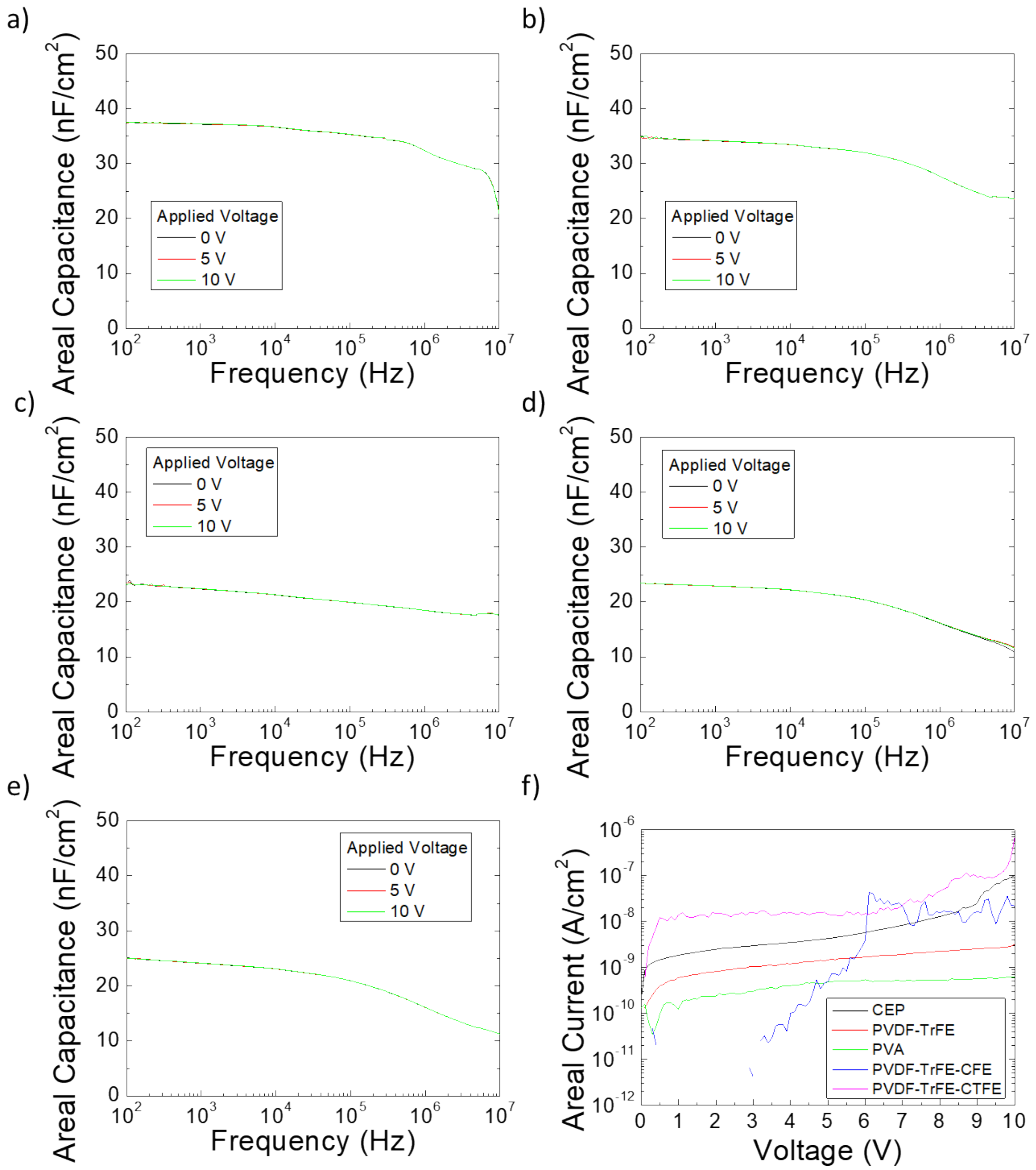

Figure S1. Capacitance-frequency measurements for MIM structures integrating multi-layer dielectric stacks fabricated with XLPS (40 nm) and with a) CEP (110 nm), b) PVDF-TrFE (160 nm), c) PVA (140 nm), d) PVDF-TrFE-CFE (200 nm) and e) PVDF-TrFE-CTFE (300 nm). f) areal leakage current for the same devices.

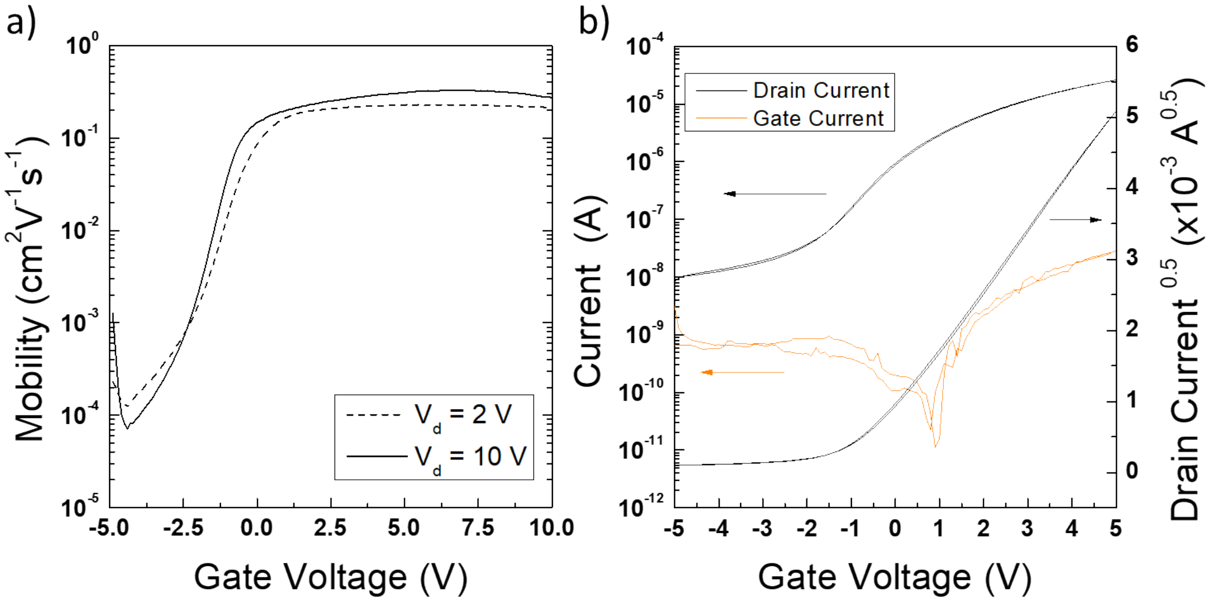

Figure S2. a) Calculated mobility in the linear and saturation regimes for an OFET (W = 2 mm, L = 10 μm) based on P(NDI2OD-T2) and our bilayer dielectric with CEP (areal capacitance C_{diel} = 32.9 nF cm^{-2}) and b) transfer curve for the same device operated with a maximum voltage bias of 5 V.

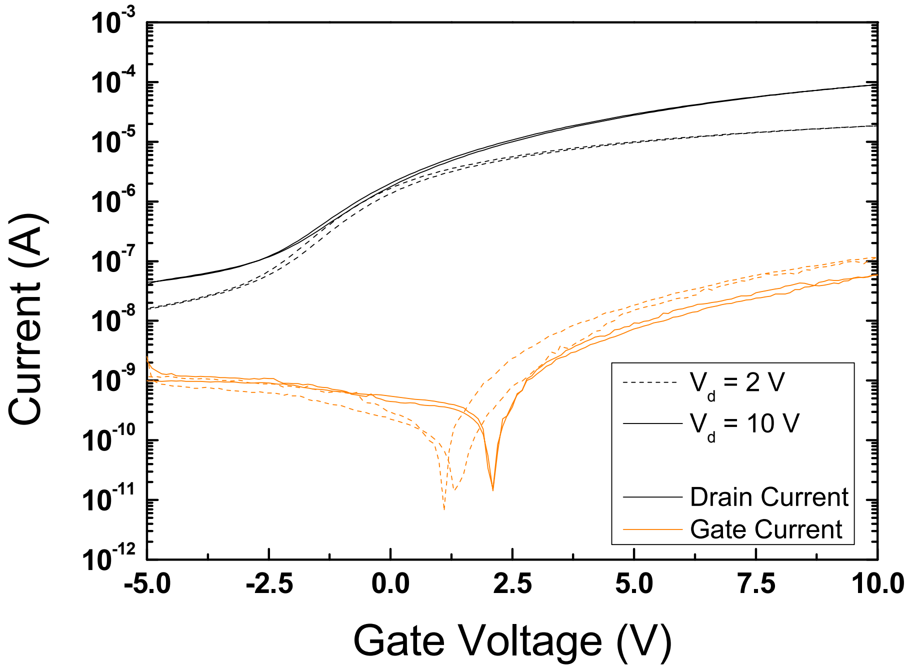

Figure S3. a) Transfer curve for an OFET (W = 2 mm, L = 10 μm) based on P(NDI2OD-T2) and our bilayer dielectric with XLPS (60 nm) and CEP (150 nm) (areal capacitance C_{diel} = 25.6 nF cm^{-2}).

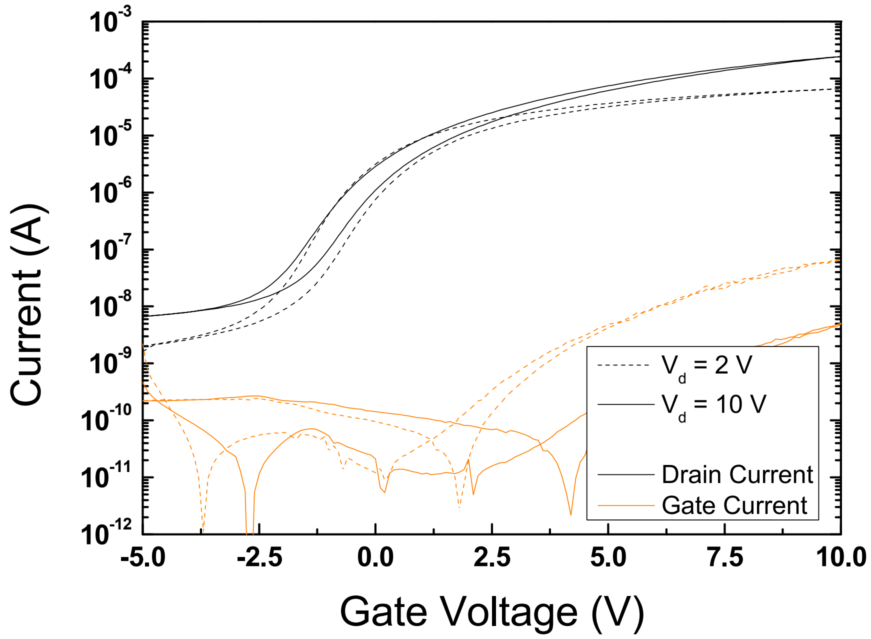

Figure S4. Transfer curve for an OFET (*W* = 2 mm, *L* = 10 µm) based on P(NDI2OD-T2) and our bilayer dielectric with XLPS (25 nm) and CEP (150 nm) (areal capacitance C_{diel} = 41.9 nF cm^{-2}).

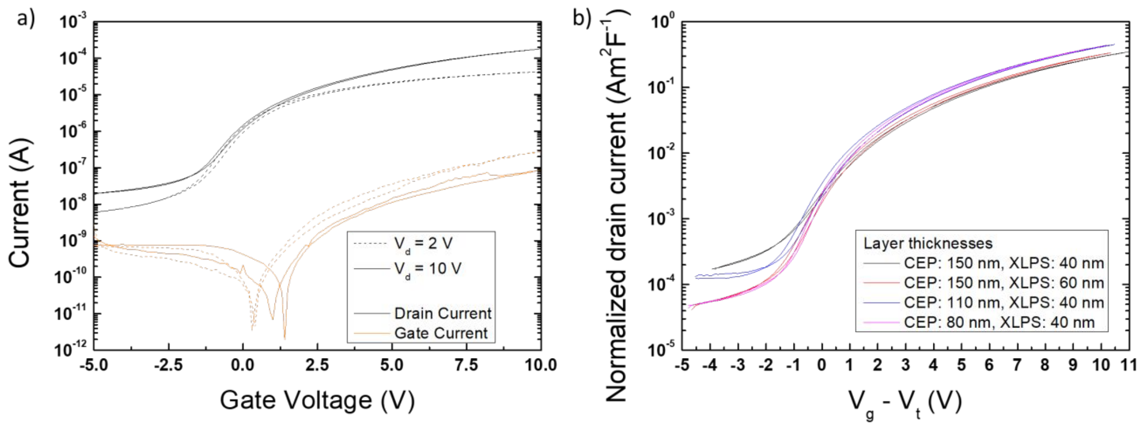

Figure S5. a) Transfer curve for the OFET (*W* = 2 mm, *L* = 10 µm) based on our bilayer dielectric with a CEP layer thickness of 80 nm (areal capacitance C_{diel} = 41.9 nF cm^{-2}) and b) superimposed capacitance-normalized transfer curves for devices with different thicknesses of the XLPS and CEP layers.

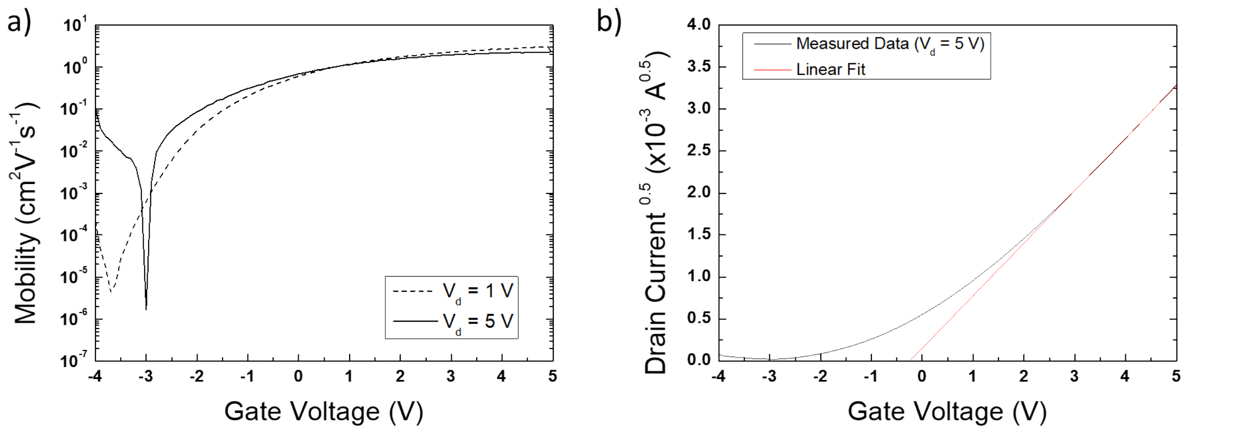

Figure S6. a) Extracted mobility for the realized OFET (W = 200 µm, L = 20 µm) based on CNTs and our bilayer dielectric with CEP and b) transfer curve (V_d = 5 V) for the same device.

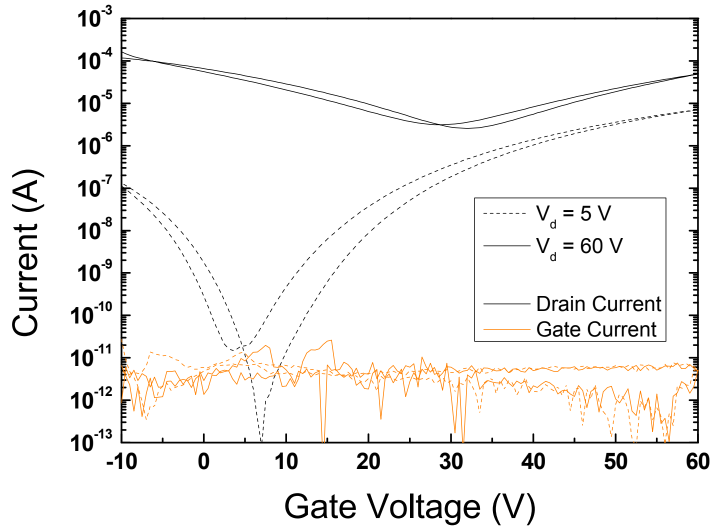

Figure S7. Transfer curve for the OFET (W = 200 µm, L = 20 µm) based on CNTs and a PMMA dielectric (thickness 550 nm, C_{diel} = 5.8 nF/cm^2).

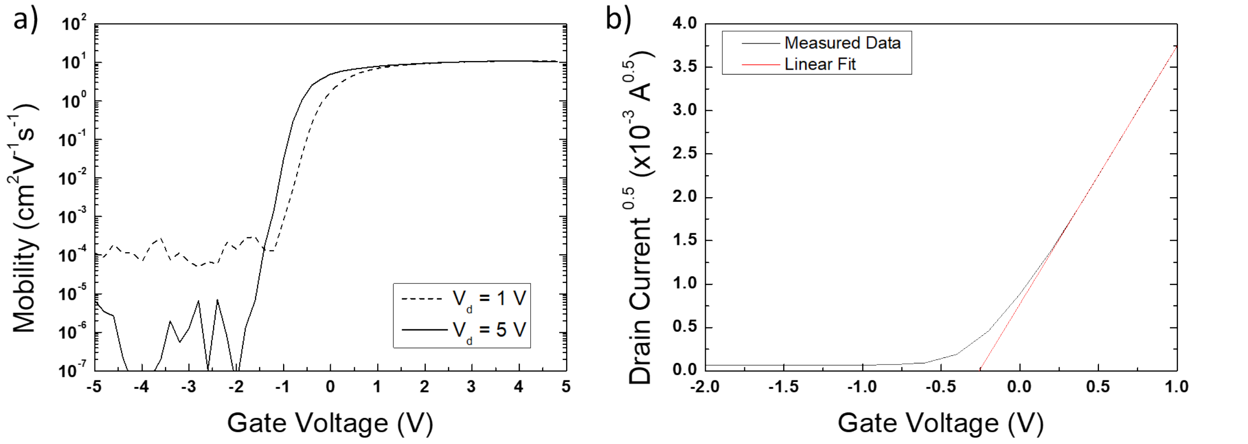

Figure S8. a) Extracted mobility for the realized OFET (W = 2 mm, L = 20 µm) based on IGZO and our bilayer dielectric with PVDF-TrFE and b) transfer curve (V_d = 1 V) for the same device.

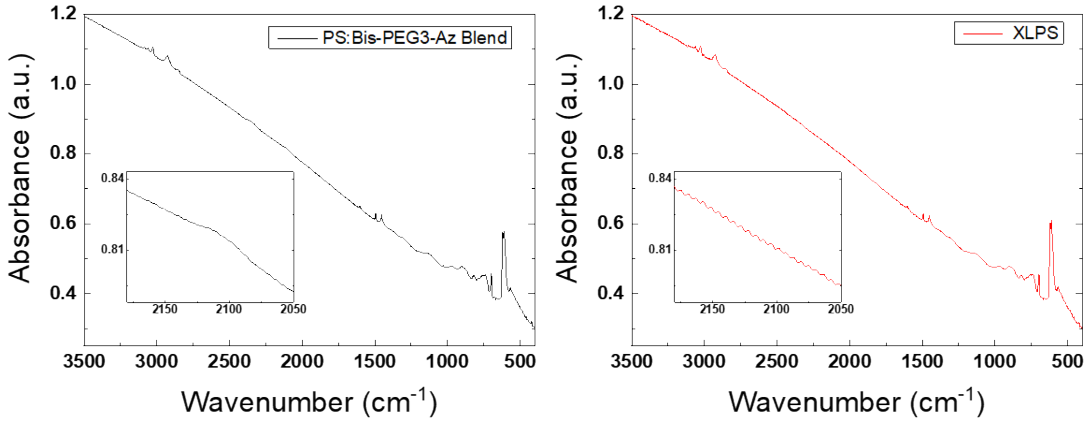

Figure S9. IR raw absorption spectra of the PS:Bis-PEG3-Azide blend (left) and of the XLPS (right).

SUPPLEMENTARY DISCUSSION

Measurement of the frequency behavior of the dielectric constant of the high-k polymers and of cross-linked PS.

The dielectric constant of the selected high-k polymers and of cross-linked PS (XLPS) was extracted by measuring the capacitance of metal-insulator-metal (MIM) capacitors (Figure SD1) with an Agilent 4294A Impedance Analyzer.

The capacitance (*C*) is:

$$C = \frac{\varepsilon_0 \varepsilon_r}{t}$$

with ε_0 and ε_r the vacuum permittivity and the relative permittivity of the layer and *t* is the thickness of the layer.

For multi-layer dielectrics, the experimental data fit well with the theortetical capacitance calculated based on the model of two capacitors in series:

$$C_{die} = \left(C_{XLPS}^{-1} + C_{high\,k\,layer}^{-1}\right)^{-1}$$

From which:

$$\frac{\varepsilon_{die}}{t_{die}} = \left(\varepsilon_{XLPS}^{-1}\, t_{XLPS} + \varepsilon_{high\,k\,layer}^{-1}\, t_{high\,k\,layer}\right)^{-1}$$

Which, for instance, for an XLPS ($\varepsilon_r = 2.6$) layer of 40 nm and a CEP ($\varepsilon_r = 13$) layer of 110 nm gives a total ε_{die} of ~6.3 and a C_{die} of ~37.2 nFcm^{-2}. The experimental calculated value of the same multi-layer is 37.5 nFcm^{-2} (see Figure S1 and Table 1).

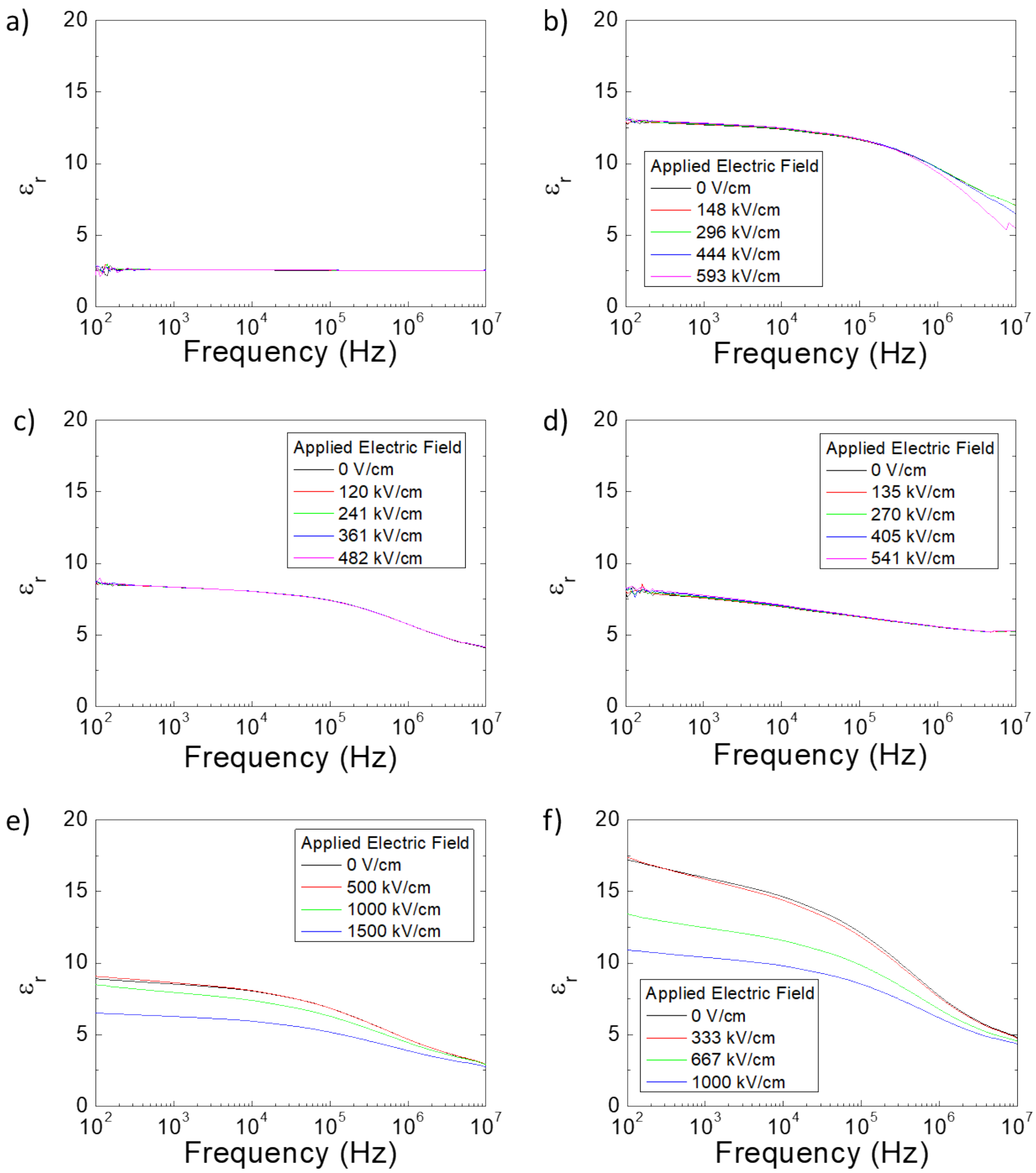

Figure SD1: Extracted ε_r vs. frequency for a) XLPS (600 nm), b) CEP (675 nm), c) PVDF-TrFE (830 nm), d) PVA (740 nm), e) PVDF-TrFE-CFE (200 nm), f) PVDF-TrFE-CTFE (300 nm). The different colors refer to different applied DC bias across the capacitor.

The results confirm that the crosslinking process for PS does not introduce non-idealities in the ε_r-f curve, which remains ideally flat across the range of interest for frequencies and applied electric field. Moreover, the measured $\varepsilon_r = 2.6$ does not deviate from pristine PS.

All the high-k polymers exhibit dielectric relaxation above their respective relaxation frequencies, but some of them (in particular CEP, PVDF-TrFE, PVDF-TrFE-CFE) remain reasonably flat before the relaxation frequency, identifying an operational frequency range for the near-ideal operation of FET devices. Moreover, the majority of the investigated materials do not exhibit significant variation of ε_r (due to *e.g.* ferroelectric effects) below 500 kV/cm, which is the range of interest for the applied electric field.

The normalization of the extracted ε_r to the value at a frequency of 1 kHz allows to determine the polymer with the flattest frequency response and the highest relaxation frequency (Figure SD2). By defining the relaxation frequency as the frequency where ε_r is attenuated by 3 dB with respect to its

value at 1 kHz, we have for CEP the best combination between a relaxation frequency of 1.73 MHz and the most reduced rolloff slope at lower frequency.

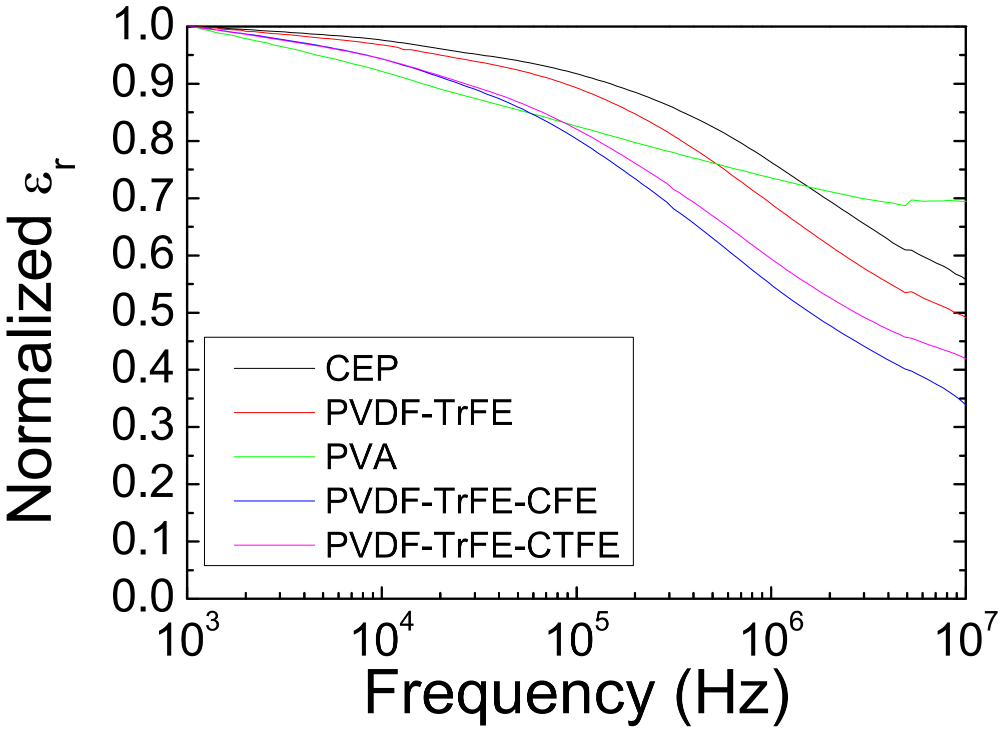

Figure SD2: Normalized ε_r vs. frequency for CEP, PVDF-TrFE, PVA, PVDF-TrFE-CFE and PVDF-TrFE-CTFE.

Measurement of the magnitude of the electric field in the XLPS and CEP dielectric layers, within multilayer dielectric stacks of different thickness.

For bilayer capacitors:

$$\begin{cases} \varepsilon_{CEP}E_{CEP} = \varepsilon_{XLPS}E_{XLPS} \\ E_{CEP}t_{CEP} + E_{XLPS}t_{XLPS} = V \end{cases}$$

where the subscripts *CEP* and *XLPS* refer to the respective materials, E is the magnitude of the electric field, V is the voltage across the multilayer structure, t is the layer thickness, ε is the relative dielectric constant. With the measured dielectric constants $\varepsilon_{XLPS} = 2.6$ and $\varepsilon_{CEP} = 13$ (Figure SD1), with $V = 10$ V and for different thicknesses of the layers we calculated:

t_{XLPS} [nm]	t_{CEP} [nm]	E_{XLPS} [V/cm]	E_{CEP} [V/cm]
60	150	1,111,111	222,222
40	150	1,428,571	285,714
25	150	1,818,182	363,636
40	110	1,612,903	322,581
40	80	1,785,714	357,143

Table SD1: Magnitude of E_{XLPS} and of E_{CEP} for different thicknesses of the dielectric layers in the multilayer stack.

AFM analysis of crosslinked polystyrene surface topography before and after solvent washing

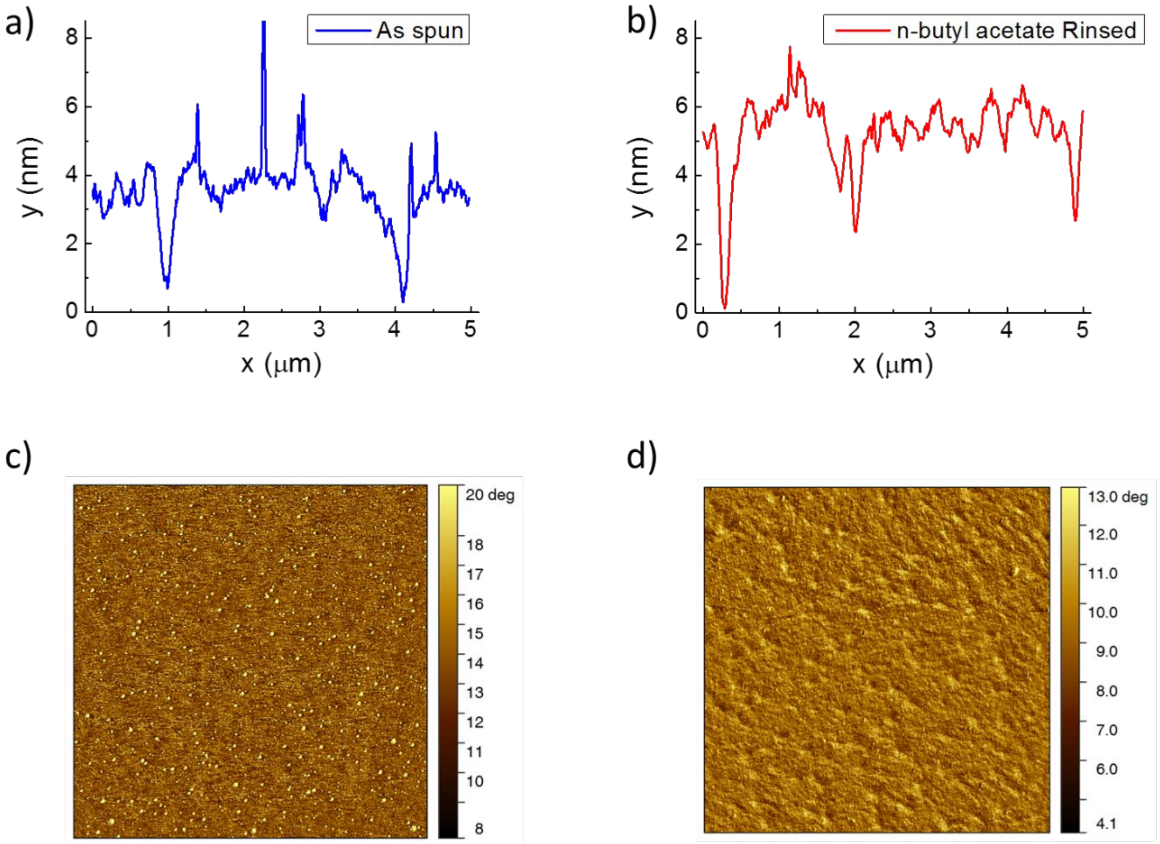

Figure SD3: a-b) Profile analysis of the dielectric layer as spun (a) and after rinsing with n-butyl acetate (b). **c-d)** AFM phase maps of the analyzed surfaces before (c) and after (d) rinsing.

In order to exclude the possibility of pass-through pinholes in the cross-linked films, further analysis was carried out on the AFM maps of the dielectric layers. Both the as-spun and the rinsed samples have average surface roughness (R_{rms}) below 1 nm. Some slight changes are present. Specifically, prior to washing, some particle-like phases are present on top of the PS-crosslinked (XLPS) film, which are probably related to some aggregated Bis-PEG3-Az phases. We infer that the absence of these particles in the washed films are associated with the removal of such Bis-PEG3-Az aggregates. Importantly, both the as-spun and the rinsed samples present valleys with an average depth of 3.4 nm and maximum depth of 5 nm (Fig SD3 a,b). Considering the width of the observed depressions (≈ 150 nm) and the nominal apex radius of the tip used (<10 nm), such small variations in thickness cannot be ascribed to pass-through holes in a 40 nm film. In addition, the phase images (Fig. SD3 c,d) do not display any sharp contrast over the whole probed surface. If deep pinholes were present, the tip would not be able to touch the surface while scanning in correspondence of a deep pass-through hole, thus inducing a sudden jump in the phase.
